# Comparison between better and poorly differentiated locally advanced gastric cancer in preoperative chemotherapy: a retrospective, comparative study at a single tertiary care institute

**DOI:** 10.1186/1477-7819-12-280

**Published:** 2014-09-08

**Authors:** Li-Bo Sun, Guo-Jie Zhao, Da-Yong Ding, Bin Song, Rui-Zhi Hou, Yong-Chao Li

**Affiliations:** >Department of Gastrointestinal Surgery, China-Japan Union Hospital, Jilin University, No. 126, Xiantai Street, Changchun, 130033 China

**Keywords:** locally advanced gastric cancer, pathological differentiation, preoperative chemotherapy, surgery

## Abstract

**Background:**

Gastric cancer is the third leading cause of cancer-related mortality in China, and the long-term survival for locally advanced gastric cancer is very poor. Simple surgery cannot yield an ideal result because of the high recurrence rate after tumor resection. Preoperative chemotherapy could help to reduce tumor volume, improve the R0 resection rate (no residual tumor after surgery), and decrease the risk of local tumor recurrence. The aim of this study was to evaluate the influence of pathological differentiation in the effect of preoperative chemotherapy for patients with locally advanced gastric cancer.

**Methods:**

Patients with locally advanced gastric cancer (*n* = 32) received preoperative chemotherapy under the XELOX (capecitabine plus oxaliplatin) regimen. According to pathological examination, patients’ tumors were classified into better (well and moderate) and poorly differentiated (lower differentiated and undifferentiated) groups, and the clinical response rate, type of gastrectomy, and negative tumor residual rate were compared between the two groups of patients. Morphological changes and toxic reactions were monitored after chemotherapy.

**Results:**

The results showed that the clinical response rate in the better differentiated group was significantly higher than that in the poorly differentiated group (100% versus 25%, *P* = 0.000). The partial gastrectomy rate in the better differentiated group was significantly higher than that in the poorly differentiated group (87.5% versus 25% *P* = 0.000). A significant shrinking of tumor and necrosis of tumor tissues caused by chemotherapy could be observed.

**Conclusions:**

In conclusion, the better differentiated group with locally advanced gastric cancer is suitable for preoperative chemotherapy under the XELOX regimen, and as a result of effective preoperative chemotherapy, much more gastric tissue can be preserved for the better differentiated group.

## Background

Gastric cancer is one of the most common malignancies in Asia, especially in China, Korea, and Japan [1]. It is the third leading cause of cancer-related mortality in China, and Chinese patients with gastric cancer account for 42% of the worldwide patient population with gastric cancer [2]. Surgical resection of the tumor is the most effective approach in increasing the long-term survival of patients with early stage gastric cancer [3]. The five-year survival rate of patients with resectable gastric cancer in advanced stages (stages III or IV) can be improved through combined surgical management with perioperative chemotherapy [4]. The benefits of preoperative chemotherapy (neo-adjuvant chemotherapy) for patients with gastric cancer are as follows: reduces tumor volume, which results in tumor downstage, improves the R0 resection rate (no residual tumor after surgery), acts on micrometastasis, decreases the risk of local tumor recurrence, and aids in evaluating tumor chemosensitivity to cytotoxic drugs [5–10]. In locally advanced gastric cancer, the primary tumor is invaded through the submucosal layers of gastric tissues, with regional nodal involvement, and occupies most of the normal gastric cell lines [11, 12]. Although the long-term effects remain controversial, preoperative chemotherapy for locally advanced gastric cancer has shown encouraging rates of pathologic complete response and R0 resection, with acceptable rates of acute and late toxicities [13, 14]. However, no report was found on the factor of pathological differentiation in preoperative chemotherapy of locally advanced gastric cancer. Therefore, the aim of this study was to evaluate the influence of pathological differentiation in the effect of preoperative chemotherapy for patients with locally advanced gastric cancer.

In this study, we compared the clinical response rate of preoperative chemotherapy between better and poorly differentiated locally advanced gastric cancer, and discussed its effect in the preservation of gastric tissue during gastrectomy.

## Methods

### Patients

Patients who had received preoperative chemotherapy and surgical treatment for locally advanced gastric cancer in the gastrointestinal department of the China-Japan Union Hospital of Jilin University, China, between April 2009 and March 2013, were retrospectively reviewed. The preoperative diagnosis was made through endoscopy, biopsy, endoscopic ultrasound, and enhanced computed tomography. The cancer staging was evaluated according to the Union for International Cancer Control tumor-node-metastasis classification (sixth edition) [15]. Patients were fully informed about the side effects of preoperative chemotherapy and surgery, and they chose this treatment by themselves voluntarily. Preoperative chemotherapy for locally advanced gastric cancer was approved by the Medical Ethics Committee of the China-Japan Union Hospital of Jilin University, China.

### Preoperative chemotherapy and surgery

As preoperative chemotherapy, the XELOX (capecitabine plus oxaliplatin) regimen was used in this study [14], as follows: intravenous infusion of oxaliplatin 130 mg/m^2^ over 2 hours on Day 1, followed by capecitabine 1,000 mg/m^2^ orally twice daily for 2 weeks. This cycle was repeated once every 3 weeks, and the patients were given two cycles before evaluation of the chemotherapeutic effect. Clinical efficacy was evaluated by computed tomography and endoscopy. Patients with resectable tumors after chemotherapy were chosen for surgery. Patients with unresectable tumors after two cycles of chemotherapy continued to receive chemotherapy (for a total of four cycles), after which the efficacy was again evaluated by computed tomography and endoscopy. At this point, patients with resectable tumors would undergo surgery, and patients with unresectable tumors would be excluded from the study. Some patients whose tumors were resectable initially, but for whom total gastrectomy seemed unavoidable were also included in this study. The choice of surgical type depended on the treating surgeon’s preference, primary tumor location, and extent of disease.

### Evaluation for efficacy and adverse events monitoring

The tumors’ reaction to prechemotherapy was evaluated as follows: (1) complete response, complete disappearance of the tumor; (2) partial response, a decrease of more than 30% in tumor size; (3) progressive disease, tumor size increased, more than 20%; and (4) stable disease, no change found in tumor size. The clinical response rate was calculated as follows:


Repeated computed tomography was used to evaluate the change in size of the metastasizing lymph node, and repeated endoscopy examination was used to evaluate changes in the primary gastric carcinoma. Patients’ liver and kidney function, bone marrow hematopoiesis, gastrointestinal reactions, and related adverse events were closely monitored during the treatment. Toxic reactions were evaluated using the National Cancer Institute Common Toxicity Criteria (version 3.0) [14].

### Statistical analysis

The patient’s age and body mass index were presented as χ ± standard deviation. The chi-square test was used to compare the differences in clinical response rate, operation type, and R0 resection rate. All statistical analyses were performed using SPSS software, version 11.0 (SPSS Inc, Chicago, United States).

## Results

### Patients’ characteristics

A total of 32 patients with locally advanced gastric cancer were enrolled in this study. The patients’ characteristics (age, sex, and body mass index (kg/m^2^)), pathological degree of differentiation, and preoperative pathological stages are listed in the Table 1.

**Table 1:** **Patients’ general characteristics**

	Better differentiated group (***n***= 16)	Poorly differentiated group (***n***= 16)	***P***
Male:female	9:7	11:5	0.465
Average age (years)	53.80 ± 4.21	52.50 ± 4.67	0.522
Average body mass index	35.24 ± 5.35	36.10 ± 7.23	0.705
Degree of differentiation:			
Well	4		
Moderate	12		
Lower		6	
Undifferentiated		10	
Preoperative tumor-node-metastasis stage:			
III	12	13	0.669
IV	4	3	0.059
Mean number of chemotherapy cycles	2.8 ± 0.75	3.4 ± 0.50	

### Response rate of preoperative chemotherapy

Of the 32 patients, no patient was rated as having complete response or progressive disease, 20 patients were rated as having a partial response, and 12 patients were rated as having stable disease. The overall clinical response rate was 62.5%. Altogether, 26 patients (81.3%) received surgical R0 resection. However, six (37.5%) tumors with poor differentiation were considered as unresectable after four cycles of preoperative chemotherapy.

### Morphological changes after preoperative chemotherapy

**Figure 1:**
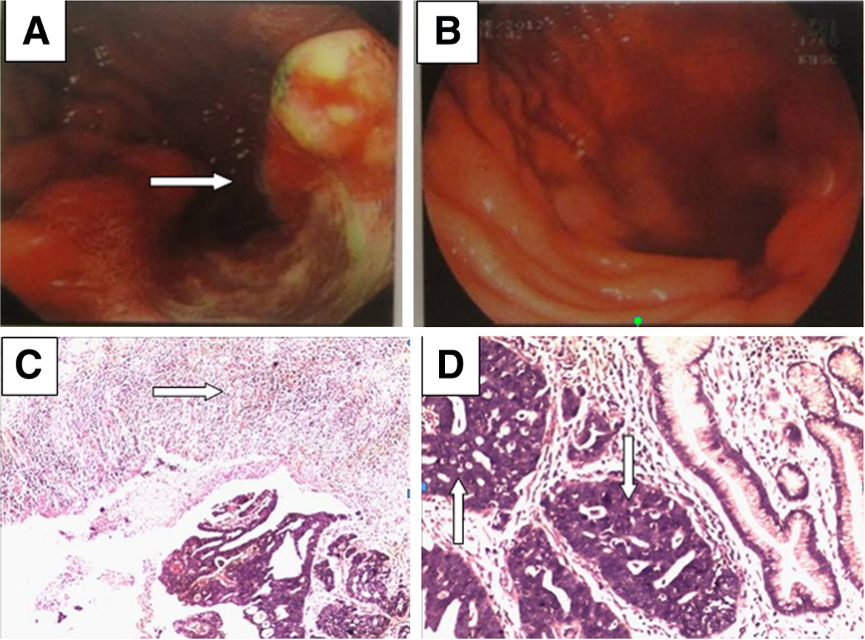
**Morphological changes of local advanced gastric cancer before and after preoperative chemotherapy. (A)** Gross gastric carcinoma (endoscopy view) before chemotherapy. **(B)** Obviously shrunk gastric carcinoma (endoscopy view) after chemotherapy in the same patient. **(C)** Tumor tissue surrounded by inflammatory tissue (arrow) after chemotherapy. H & E staining, original magnification ×40. (**D**) Gastric cancer cells showed obvious nucleus necrosis after chemotherapy. H & E staining, original magnification ×100.

Figures 1A and 1B show a significant change in tumor size (partial response) before and after preoperative chemotherapy. Figures 1C and 1D show the necrosis of tumor tissue surrounded by inflammatory tissue after preoperative chemotherapy.

### Comparison of response and surgical results between different pathological differentiations

The pathological differentiation was classified into two groups: better differentiated (well and moderately differentiated) and poorly differentiated (lower differentiated and undifferentiated). No difference was found in the age, sex, body mass index, tumor-node-metastasis stage, and tumor size between the two groups. The clinical response rate in the better differentiated group was significantly higher than that in the poorly differentiated group. The partial gastrectomy rate in the better differentiated group was significantly higher than that in the poorly differentiated group (Table 2). Neither death nor severe complication occurred in this study. No difference was found in surgical time and incidence rate of postoperative complications between the well and poorly differentiated groups.

**Table 2:** **Comparison of chemotherapy results between better and poorly differentiated groups**

	Better differentiated group (***n***= 16)	Poorly differentiated group (***n***= 16)	***P***
Clinical reaction rate	100% (16/16)	25% (4/16)	0.000
Partial gastrectomy	87.5% (14/16)	25% (4/16)	0.000
Total gastrectomy	12.5% (2/16)	37.5% (6/16)	0.654
Unresectable cases			
Prechemotherapy	18.7% (3/16)	62.5% (10/16)	0.012
Postchemotherapy	0 (0/16)	37.5% (6/16)	0.024
R0 resection rate	100% (16/16)	62.5% (10/16)	0.018
Toxicity reaction rate	100% (16/16)	100% (16/16)	

R0, no residual tumor after surgery.

### Toxicity

No treatment termination or death occurred as a result of a toxic reaction. During preoperative chemotherapy, there were different degrees of toxic and adverse reactions, mainly myelosuppression, liver dysfunction, and gastrointestinal reactions. Toxic reaction occurred in all the patients receiving preoperative chemotherapy, to different extents. Leukopenia (*n* = 24; 75%) was the most commonly reported adverse reaction. As markers of liver injury, alanine aminotransferase and aspartate aminotransferase levels were increased in nine (28.1%) and eight patients (25%), respectively. Nausea and vomiting were the commonest digestive-tract reactions, with 19 patients (59.3%) reporting vomiting, and 25 patients (67.7%) reporting nausea. Toxic neurological reaction occurred in 21 patients (78.1%). The toxic reactions reported in this study were similar with previous reports [14, 16].

## Discussion

Unlike early gastric cancer, surgical treatment of advanced gastric cancer is not satisfactory because of tumor local invasion and severe lymph node metastasis, with a survival time of no longer than one year [17, 18]. However, surgery is still the primary treatment modality for achieving a potential cure and can be beneficial in the palliation of advanced gastric cancer [19, 20]. The high recurrence rate after surgical resection for locally advanced gastric cancer was considered the main reason for poor treatment results [21, 22]. Many clinical studies have shown that chemotherapy can downstage the tumor, eliminate micrometastasis, and make some unresectable gastric cancers resectable, thereby prolonging the survival time of patients [23–26].

Recently, the XELOX regimen has been used as a new chemotherapeutic strategy for locally advanced gastric cancer patients, which was easier to accept in clinical practice, with an encouraging 63% clinical response rate and a median survival time of 11.9 months [27]. In the present study, similar results were obtained, with a clinical response rate of 62.5% and 81.3% R0 resection. On comparing the clinical response rate between the better differentiated and poorly differentiated groups, it was found that the better differentiated group showed a 100% clinical response rate, whereas the poorly differentiated group showed only 25%. This result strongly suggested that well and moderately differentiated locally advanced gastric cancer is a candidate for preoperative chemotherapy. Moreover, total gastrectomy could be avoided in patients with well differentiated gastric cancer, since the recovery of normal gastric tissue was a result of effective preoperative chemotherapy. Although the short-term results for better differentiated locally advanced gastric cancer were promising in this study, longer survival times need to be observed further.

After chemotherapy, well differentiated larger tumors were found to be obviously shrunken. After H & E staining, the necrosis of tumor tissues was easily seen under a microscope, commonly surrounded by inflammatory tissue, forming a typical tissue morphology after sensitive chemotherapy. Based on the effective chemotherapy, the recovery of normal gastric tissues resulted in the possibility of preserving some stomach, other than removing the total stomach, to obtain R0 resection.

The toxic reactions to chemotherapy were very common and were the main cause of patients refusing or discontinuing chemotherapy. In the present study, patients experienced different degrees of toxic and adverse reactions, especially during the first cycle. The patients could recover from leukopenia and liver function abnormality after two to three weeks of rest. Nausea and vomiting often occurred during the intravenous infusion of oxaliplatin at the beginning of therapy, wherein liquid transfusion was necessary to keep the acid-base balance and to supply nutritional energy. For patients with severe toxic reactions, delaying the treatment was deemed necessary.

There are several limitations of this study to note. Firstly, this study was a retrospective study. Retrospective studies are inherently less robust than prospective studies. Secondly, the sample sizes of the groups were relatively small. It is possible that additional differences would emerge in a larger study. Hence, a large prospective study at multiple centers could provide more robust and generalizable information about the influence of pathological differentiation in the effect of preoperative chemotherapy for patients with locally advanced gastric cancer.

## Conclusions

From this study, it is concluded that better differentiated locally advanced gastric cancer is suitable for preoperative chemotherapy under the XELOX regimen. As a result of effective preoperative chemotherapy, much more gastric tissue can be preserved in patients with better differentiated locally advanced gastric cancer.

## Abbreviations

H & E: hematoxylin and eosin, R0 resection, no residual tumor after surgery.
